# Dosimetric evaluation of a spinal cord dose‐limiting 3D‐CRT technique for radiotherapy of spinal metastases

**DOI:** 10.1002/acm2.14042

**Published:** 2023-09-07

**Authors:** Christian Cornelius Arnold, André Toussaint, Frederick Mantel, Michael Flentje, Klaus Bratengeier

**Affiliations:** ^1^ Department of Radiotherapy and Oncology Goethe University Frankfurt Frankfurt Germany; ^2^ Department of Radiation Oncology University of Wuerzburg Wuerzburg Germany

**Keywords:** 3D‐CRT, palliative radiotherapy, planning study, re‐irradiation, spinal metastases

## Abstract

**Background:**

To evaluate the possible advantages of a simple spinal cord (SC) dose‐limiting three‐dimensional conformal radiotherapy (3D‐CRT) technique in comparison to conventional two‐dimensional (2D) techniques and other 3D‐CRT techniques for spinal bone irradiation.

**Methods:**

For 41 spinal target volumes, seven different techniques were evaluated, using a standard schedule of 30 Gy in 10 fractions. The SC dose‐limiting 3D‐CRT technique *1F2S‐18MV* using a single posterior field (F) supplemented by two anterior segment fields (S) and 18‐MV photon beams was compared to two conventional 2D techniques (a single posterior field, *PA*, and two opposed anterior‐posterior fields, *APPA*), three other 3D‐CRT techniques (a single posterior field supplemented by four segment fields, *1F4S*; two wedged fields, *WD*, and the SC dose‐limiting variant using 6 MV, *1F2S‐6MV*) along with the original clinically applied plans.

**Results:**

1F2S‐18MV demonstrated notably better results for all target volume parameters compared to the conventional 2D techniques (*p* < 0.001). Limitation of the SC dose was significantly superior with 1F2S‐18MV in comparison to PA and APPA (SC Dmean: 28.9 ± 0.4  vs. 30.1 ± 0.6 Gy and 30.1 ± 0.4 Gy; SC Dmax: 30.9 ± 0.7  vs. 32.5 ± 1.0 Gy and 31.8 ± 0.7 Gy; SC D1cm^3^: 30.1 ± 0.6  vs. 31.7 ± 0.9 Gy and 31.1 ± 0.6 Gy; *p* < 0.001). Likewise, lower mean SC doses with 1F2S‐18MV were observed in comparison to the more treatment time‐consuming 3D‐CRT techniques (1F4S, WD) and the original plans without relevant compromises on the dose homogeneity in the target volume and the dose exposure to the other OARs.

**Conclusion:**

In treatment planning of spinal metastases, simple variants of 3D‐CRT‐techniques like 1F2S‐18MV can offer a significant dose limitation to the SC while providing a sufficient dose coverage of the target volume. Especially in patients with favorable life expectancy and potential need for re‐irradiation, such SC dose‐limiting 3D‐CRT techniques may be a reasonable approach.

## 1 INTRODUCTION

Bone metastasis is a challenging disease, in which the spine is the main site of manifestation.^1^ Radiotherapy (RT) of bone and, notably, spinal metastases is an effective treatment option to achieve pain relief and local control as well as alleviating neurological symptoms.^2^, ^3^, ^4^

Despite the growing use of intensity‐modulated (IMRT) and stereotactic body radiotherapy (SBRT), the applied techniques for irradiation of the spine in worldwide everyday clinical practice still often include the use of straightforward conventional techniques (e.g., a single posterior‐anterior field, *PA*).^5^ Although proven effective, these techniques might deliver an uncertain and high dose to the spinal cord (SC).^6^, ^7^ In regard to the increasing survival rates of cancer patients with metastatic disease and the potential need for retreatment of the spine, the presumed high SC doses of these simple 2D conventional techniques might be an avoidable disadvantage.^8^

The advancing implementation of highly precise and resource‐consuming SBRT or IMRT for selected patients might counteract this clinical practice of using unsophisticated techniques in a predominant proportion of cases.^9^, ^10^ After all, the utilization of three‐dimensional conformal radiotherapy (3D‐CRT) methods gives radiation oncologists the opportunity to treat patients with techniques that claim to be a middle ground of precision. In fact, the usage of 3D‐CRT for the treatment of spine metastases might be standard of care in many centers. However, in literature, potential advantages of straightforward planned 3D‐CRT techniques have not yet been thoroughly evaluated. To our knowledge, only two small studies exclusively addressed the comparison of conventional two‐dimensional (2D) and 3D‐CRT‐techniques in spinal irradiation thus far.^6^, ^7^ Hence, we conducted a planning study to evaluate this matter by comparing dose parameters of several conventional 2D and 3D‐CRT techniques for spine irradiation. One 3D‐CRT technique (1F2S‐18MV) was designed to limit the dose to the SC and should therefore stand in the center of the examination.

## 2 METHODS

### 2.1 Volumes of interest

Forty‐one spinal target volumes of 34 patients were included in the study. All patients had been irradiated in the affected localization prior to study inclusion from 2012 to 2014. Target volumes with radiographical (suspected) tumor involvement of the spinal canal were excluded. The target volumes encompassed 1–12 (median: 4) vertebrae. All vertebral levels of the spinal column from the first cervical to the fifth lumbar vertebra were represented (see Figure S1). As the common practice in our hospital, the planning target volume (PTV) was directly contoured in corresponding computer tomography (CT) slices by a specialist physician and included all bone structures of the involved vertebrae plus safety margin. Superior and inferior borders of the PTV were limited to the intervertebral spaces. A clinical target volume (CTV) was retrospectively generated by subtracting a 0.5 cm margin from the PTV.

Organs of risk (OARs) were delineated on relevant CT‐slices and included the parotid gland (*n* = 8), the posterior wall of the pharynx (*n* = 13), the larynx (*n* = 13), the esophagus (*n* = 22), the heart (*n* = 15), the lungs (*n* = 19), the liver (*n* = 13), the small intestine (*n* = 11), the left kidney (*n* = 13), the right kidney (*n* = 14), and the SC (*n* = 41). SC was estimated by contouring the spinal canal as a surrogate.

### 2.2 Treatment planning

All patients underwent planning CT scans in a stable supine position. CT scanning was carried out with a *Siemens Sensation Open CT Scanner* (SIEMENS, Erlangen, Germany) with a slice thickness of 0.3  or 0.5 cm. Treatment planning was performed with *Pinnacle 3, version 9.2* (Philips Healthcare, Eindhoven, Netherlands). The prescription dose was 30 Gy at 3 Gy per fraction. A central point in the PTV was determined as the isocenter. Device data were based on a *Synergy* Linac system (Elekta, Crawley, UK) with a multileaf collimator (MLC) leaf width of 0.5 cm (Elekta Agility, Crawley, UK). The prescribed dose was normalized to the mean value in the CTV. Inhomogeneity corrections were taken into account by the Collapsed Cone dose algorithm. Dose calculation grid size was 0.3 cm.

In addition to the original 3D‐CRT reference plans (which had been individually used for actual treatment), six different treatment plans were created for each target volume: Two conventional techniques (PA and APPA) and four 3D‐CRT techniques (named 1F2S‐18MV, 1F2S‐6MV, 1F4S, and WD). Eighteen MV photon beams were used in all techniques with the exception of 1F2S‐6MV.

**FIGURE 1:**
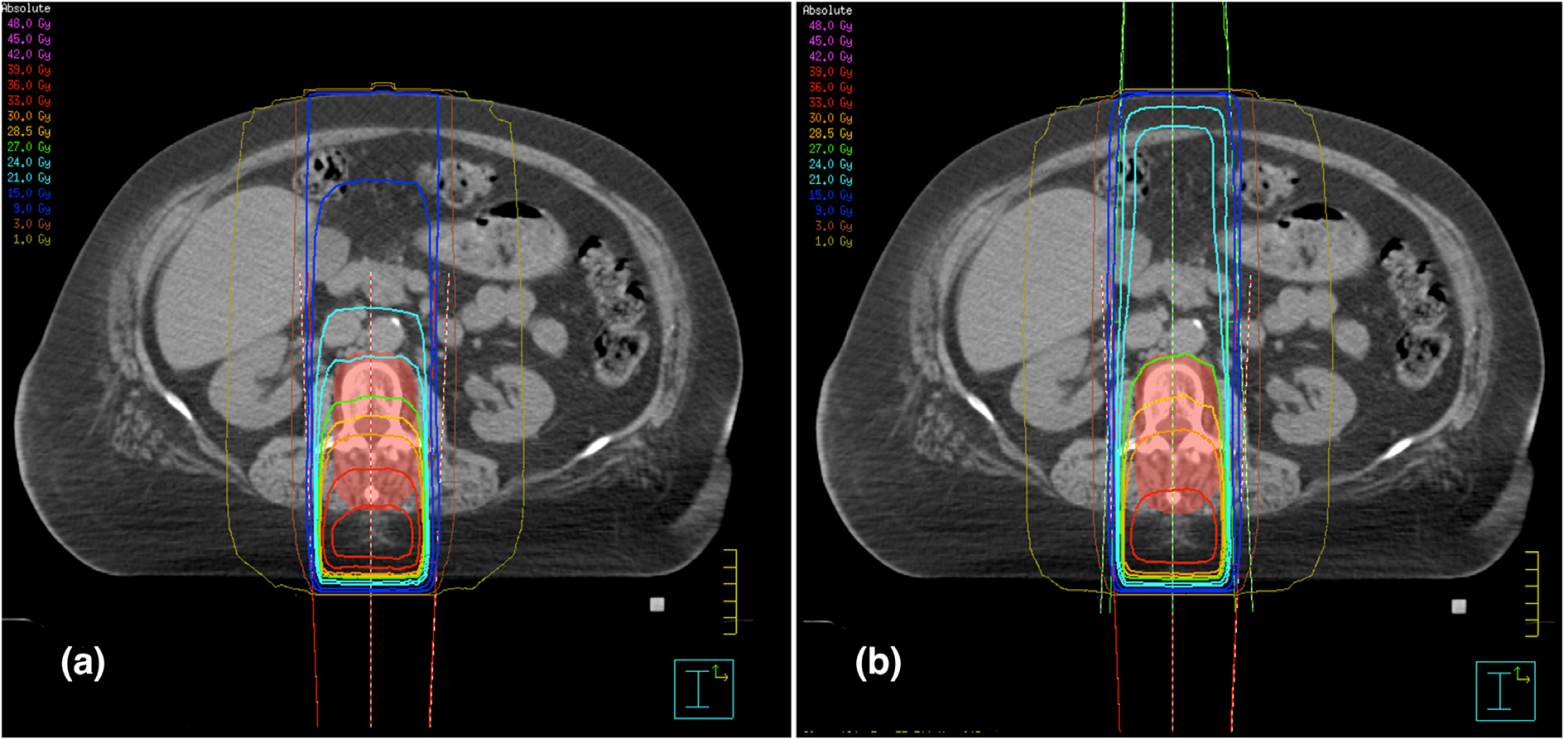
Representative axial slices for the PA (a) and APPA (b) techniques showing dose distributions. The PTV is indicated as the red shaded area.

**FIGURE 2:**
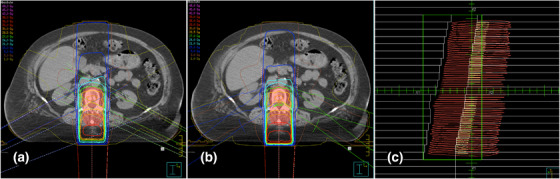
1F2S‐18MV (a) and 1F2S‐6MV (b) techniques with exemplary segment field in beam's eye view (c). Representative axial slices with dose distributions are shown for 1F2S‐18MV and 1F2S‐6MV plans. The MLC aperture for the lateral oblique segment field displayed in beam's eye view is adapted to limit dose exposure to the SC. The PTV is indicated as the red shaded area.

**FIGURE 3:**
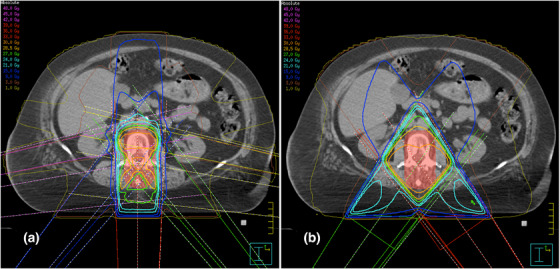
Representative axial slices for the 1F4S (a) and WD (b) techniques showing dose distributions. The PTV is indicated as the red shaded area.

PA consisted of a single posterior‐anterior field with a gantry angle of 180° (Figure 1a). MLC leaves were positioned automatically around the PTV in a 0.5 cm margin laterally and 0.7 cm margin craniocaudally. In APPA plans, an opposing posterior‐anterior field with a gantry angle of 0° was added. Beam weighting in APPA was manually optimized to put the 90% isodose on a level with the ventral border of the vertebral bodies (Figure 1b). For 1F2S‐18MV, 1F2S‐6MV, and 1F4S, the single posterior field (*1F*) was supplemented by multiple smaller segment fields (*S*). In 1F2S‐18MV plans, two lateral oblique segments of 115° and 245° were added (Figure 2a). In 1F2S‐6MV plans, 6‐MV photons and a small angle rotation (±10°) around the medium gantry angles of 115° and 245° were used (Figure 2b). The small angle rotation should limit the dose in the entry areas of the segment beams. The MLC aperture of segments in 1F2S‐18MV and in 1F2S‐6MV plans was adapted to the rear edge of the vertebral bodies to limit the dose exposure of the SC (Figure 2c). In 1F4S plans, four segments were added. The MLC aperture of the lateral oblique segments (100° and 260°) and of the posterior oblique segments (140° and 220°) were adjusted to the ventral third and the ventral two thirds of the PTV, respectively (Figure 3a). Beams were weighted in order to keep the mean value of the standard deviation in the CTV as low as possible. The WD technique consisted of two posterior wedged oblique beams of 145° and 215° (Figure 3b). Weighting of the two beams was balanced (50% each).

Treatment plan delivery time was estimated using a simple formula previously described by Bratengeier et al.^11^

### 2.3 Dose parameters and statistical analysis

As target volume dose parameters, the minimum, maximum, and mean doses to the CTV, the standard deviation of the dose to the CTV as well as the minimum dose delivered to 95% of the PTV (PTVD95) were assessed. Minimum dose was defined as the dose to 100% of the respective volume and maximum dose as the dose covering 0.0 cm^3^ of the respective volume. Dose exposure to the SC was evaluated by the mean and the maximum doses to the SC and the minimum dose to the most exposed 1 cm^3^ (D1cm^3^) of the SC. Dose exposure to the OARs was assessed by the mean doses to the organ tissues as well as the maximum doses to the esophagus, the small intestine and the kidneys. Additionally, the minimum dose to the most exposed 1 cm^3^ of the larynx was analyzed. All mentioned parameters were calculated for their mean values and SPSS Statistics for Mac (version 22.0) was used for statistical comparison. For dose parameters with sample size *n* > 30 (i.e., target volume and SC parameters), mean values were considered as normally distributed and the paired‐samples *t*‐test was conducted. For dose parameters with sample size *n* < 30 (i.e., all OARs parameters except SC) test of normality were carried out first by using the Shapiro‐Wilk method. If normal distribution was assumed in this way, paired‐samples *t*‐test was conducted. In case of non‐parametric data, the Wilcoxon rank test was used. To take the risk of the multiple testing problem into account, the Bonferroni‐Holm method was applied. Additionally, pairwise comparisons were limited to comparisons of 1F2S‐18MV only since the study focused on the examination of this technique.

In the following, dose parameter values are reported as mean ± standard deviation (SD). *p*‐values of less than 0.05 are considered statistically significant. *p*‐values that are considered statistically significant by using the conservative Bonferroni‐Holm method are highlighted by asterisks.

## 3 RESULTS

Dose parameter values and p‐values are shown in Table 1. Target volume dose distribution was significantly superior in 1F2S‐18MV plans compared to PA and APPA‐plans (*p* < 0.001* each). Mean values for CTV Dmin, CTV Dmax, and PTV D95 were, respectively, 27.0 ± 1.8 , 32.7 ± 1.8, and 27.6 ± 0.6 Gy in 1F2S‐18MV plans; 24.8 ± 1.9, 34.6 ± 1.6, and 25.7 ± 0.9 Gy in PA plans; and 26.4 ± 1.7 , 33.3 ± 0.9, and 27.0 ± 0.3 Gy in APPA plans. In comparison to the more time‐consuming 3D‐CRT techniques, advantages in target volume coverage were balanced (1F2S‐18MV vs. 1F4S) or slightly inferior with 1F2S‐18MV (1F2S‐18MV vs. WD and 1F2S‐18MV vs. original plans), if at all significant.

**TABLE 1:** Dose parameter values for 1F2S‐18MV in pairwise comparison to the other techniques.^a^

Dose parameter	1F2S‐18MV	PA	*p*	APPA	*p*	1F4S	*p*	WD	*p*	Orig. pl.	*p*	1F2S‐6MV	*p*
CTV Dmin	27.0 ± 1.8	24.8 ± 1.9	<0.001*	26.4 ± 1.7	<0.001*	26.8 ± 1.9	<0.05	27.3 ± 1.9	<0.05	27.3 ± 1.4	n.s.	26.3 ± 1.3	<0.001*
CTV Dmax	32.7 ± 1.0	34.6 ± 1.6	<0.001*	33.3 ± 0.9	<0.001*	32.3 ± 0.8	<0.001*	31.6 ± 1.0	<0.001*	32.3 ± 0.8	<0.01*	34.1 ± 1.2	<0.001*
CTV Dmean	30.0 ± 0.1	30.0 ± 0.0	n.s.	30.0 ± 0.0	n.s.	30.0 ± 0.1	n.s.	30.0 ± 0.1	n.s.	30.0 ± 0.1	n.s.	30.0 ± 0.0	n.s.
SD CTV Dmean	1.0 ± 0.4	2.1 ± 0.6	<0.001*	1.4 ± 0.3	<0.001*	0.8 ± 0.3	<0.001*	0.7 ± 0.4	<0.001*	0.8 ± 0.3	<0.01*	1.4 ± 0.4	<0.001*
PTV D95	27.6 ± 0.6	25.7 ± 0.9	<0.001*	27.0 ± 0.3	<0.001*	27.5 ± 0.6	<0.001*	27.9 ± 0.8	n.s.	28.0 ± 1.0	n.s.	27.4 ± 0.8	<0.001*
SC Dmax	30.9 ± 0.7	32.5 ± 1.0	<0.001*	31.8 ± 0.7	<0.001*	31.9 ± 0.8	<0.001*	31.0 ± 1.0	n.s.	31.5 ± 0.8	<0.001*	31.1 ± 1.0	<0.01*
SC Dmean	28.9 ± 0.4	30.1 ± 0.6	<0.001*	30.1 ± 0.4	<0.001*	30.1 ± 0.3	<0.001*	29.9 ± 0.3	<0.001*	29.7 ± 2.6	<0.001*	28.5 ± 0.6	<0.001*
SC D1cm^3^	30.1 ± 0.6	31.7 ± 0.9	<0.001*	31.1 ± 0.6	<0.001*	31.3 ± 0.9	<0.001*	30.8 ± 1.0	<0.001*	32.7 ± 1.0	<0.001*	30.0 ± 0.8	<0.05
Parotid gland Dmean	3.0 ± 1.4	2.5 ± 1.4	<0.001*	2.5 ± 1.4	<0.001*	3.6 ± 1.4	<0.001*	7.9 ± 2.0	<0.001*	16.1 ± 5.2	<0.001*	3.7 ± 1.4	<0.001*
Pharynx Dmean	21.0 ± 8.6	19.3 ± 8.0	<0.001*	19.6 ± 8.1	<0.001*	20.6 ± 8.5	<0.001*	21.5 ± 8.9	<0.05	18.0 ± 8.3	<0.01	21.2 ± 8.5	<0.05
Larynx Dmean	16.6 ± 7.4	16.4 ± 7.1	n.s.	17.1 ± 7.5	n.s.	16.3 ± 7.2	<0.05	16.4 ± 8.5	n.s.	10.9 ± 6.3	<0.001*	15.9 ± 7.0	<0.01*
Larynx D1cm^3^	25.0 ± 4.5	23.4 ± 3.5	<0.01*	24.6 ± 3.6	n.s.	24.5 ± 4.3	<0.01*	26.2 ± 5.9	<0.05	21.2 ± 7.6	<0.05	24.6 ± 4.6	<0.05
Esophagus Dmax	28.5 ± 2.2	26.0 ± 1.2	<0.001*	27.3 ± 0.8	<0.001	28.2 ± 2.1	<0.05	28.6 ± 1.2	n.s.	28.1 ± 3.4	n.s.	28.1 ± 2.6	<0.05
Esophagus Dmean	12.5 ± 7.1	11.8 ± 6.8	<0.01*	12.5 ± 7.3	n.s.	12.2 ± 7.0	<0.001*	11.7 ± 6.7	<0.01	11.6 ± 6.6	<0.01*	12.2 ± 6.8	<0.05
Heart Dmean	8.1 ± 4.4	8.3 ± 4.5	<0.05	8.9 ± 5.1	<0.01*	7.9 ± 4.3	<0.001*	6.0 ± 3.4	<0.01*	6.7 ± 4.0	<0.001*	7.6 ± 4.0	<0.01*
Lungs Dmean	2.9 ± 2.1	2.4 ± 1.9	<0.001*	2.5 ± 1.9	<0.001*	2.9 ± 2.1	n.s.	4.5 ± 3.0	<0.001*	4.1 ± 2.7	<0.001*	3.1 ± 2.2	<0.001*
Liver Dmean	4.8 ± 1.9	4.3 ± 1.8	<0.001*	4.8 ± 2.0	n.s.	4.8 ± 1.8	n.s.	5.8 ± 2.1	<0.01*	5.6 ± 2.1	<0.01*	4.8 ± 1.8	n.s.
Small bowel Dmax	26.0 ± 4.7	24.1 ± 3.7	<0.01	25.9 ± 4.5	n.s.	25.9 ± 4.9	n.s.	27.2 ± 5.0	n.s.	25.7 ± 5.5	n.s.	25.7 ± 5.2	n.s.
Small bowel Dmean	3.4 ± 2.0	3.0 ± 1.8	<0.01*	3.3 ± 2.2	n.s.	3.5 ± 2.0	n.s.	4.3 ± 2.4	<0.01*	4.0 ± 2.0	<0.01	3.5 ± 2.0	n.s.
Kidney rt Dmax	25.0 ± 6.3	23.2 ± 6.2	<0.001*	23.1 ± 6.6	<0.001*	25.2 ± 6.0	n.s.	29.0 ± 3.8	<0.05	26.7 ± 3.6	n.s.	26.5 ± 5.8	<0.01*
Kidney rt Dmean	5.0 ± 2.4	2.7 ± 1.7	<0.001*	2.7 ± 1.7	<0.001*	4.5 ± 2.1	n.s.	7.9 ± 3.4	<0.001*	7.1 ± 2.5	<0.01*	6.3 ± 2.8	<0.001*
Kidney lt Dmax	23.2 ± 8.5	21.4 ± 9.0	<0.001*	21.3 ± 9.2	<0.001*	22.9 ± 7.8	n.s.	29.6 ± 3.8	<0.001	27.3 ± 3.4	<0.01	24.2 ± 8.6	<0.01
Kidney lt Dmean	4.8 ± 2.4	2.3 ± 1.5	<0.001*	2.4 ± 1.6	<0.001*	4.3 ± 2.1	<0.05	8.1 ± 3.6	<0.001*	7.3 ± 2.5	<0.01*	6.1 ± 2.7	<0.001*

Abbreviations: CTV, clinical target volume; PTV, planning target volume; PTV D95, minimum dose delivered to 95% of the PTV; SC, spinal cord; D1cm^3^, minimum dose to the most exposed 1 cm^3^; rt, right; lt, left; n.s., not significant (*p* > 0.05); Orig. Pl., original plans.

^a^ Values are shown as mean dose in Gy ± standard deviation (SD).

* *p*‐values that are considered statistically significant by using the Bonferroni–Holm method are highlighted by asterisks.

Limitation of SC dose was significantly superior with 1F2S‐18MV in comparison to PA and APPA (SC Dmean: 28.9 ± 0.4  vs. 30.1 ± 0.6 Gy and 30.1 ± 0.4 Gy; SC Dmax: 30.9 ± 0.7  vs. 32.5 ± 1.0 Gy and 31.8 ± 0.7 Gy; SC D1cm^3^: 30.1 ± 0.6  vs. 31.7 ± 0.9 Gy and 31.1 ± 0.6 Gy; *p* < 0.001*). Significantly lower mean SC dose parameter values with 1F2S‐18MV were also observed in comparison to 1F4S, WD, and the original plans (*p* < 0.001*).

Regarding the dose exposure of the other OARs, mean values of 1F2S‐18MV, PA, APPA, and 1F4S were on a similar level. As it was to be expected, lowest values for the OARs anterolateral of the PTV were achieved with PA (however, under acceptance of insufficient coverage of the target volume, as seen above). Clearly, higher dose burdens to the parotid gland and the kidneys were shown in WD‐plans, without relevant differences in the values of the other OARs parameters. Strong distinctions in the cervical area, compared to 1F2S‐18MV, were seen in dose values of the individual original plans: Because of the common use of lateral fields in this location, dose exposure to the parotid gland was relatively high, while delivering clear dose limitations to the pharynx and larynx.

Compared to the variant using 6MV (1F2S‐6MV), 1F2S‐18MV plans showed overall better results but without relevant differences.

Calculated plan delivery time for 1F2S‐18MV was 103 s, which was midway between the shortest (PA: 42 s) and longest (WD: 150 s) mean values. All times are listed in Table S1.

## 4 DISCUSSION

Due to its widely proven efficacy, RT of osseous and, in particular, spinal metastases has been part of oncological treatment regimes for decades.^12^, ^13^ In such palliative treatment settings, conventional PA and APPA techniques still play an important role, despite the overall increasing use of (much) more complex techniques.^14^, ^15^ A growing body of evidence suggests that SBRT constitutes a superior alternative to conventional RT in selected cases.^16^, ^17^, ^18^, ^19^ However, access to these highly sophisticated but resource‐consuming techniques cannot yet be provided to every patient. Hence, for the time being, conventional dosage and use of 2D (i.e., PA/APPA) and simple 3D‐CRT techniques might remain the standard of care. However, the distinction between 2D and—in terms of OAR dose limitation—potentially superior 3D‐CRT techniques has been underreported until now. We tried to fill this gap by carrying out the present planning study for the treatment of spinal metastases.

Our data indicate that simple 3D‐CRT techniques offer several benefits in terms of dose distribution in comparison to conventional 2D techniques. These findings are in line with previous data from Soyfer et al. and Yeo et al.^6^, ^7^ In those smaller series, 3D‐CRT techniques were compared to PA and APPA in ten lumbar spine and ten thoracic spine cases, respectively. More homogenous dose distribution of the target volume with 3D‐CRT and overall better dose limitation to the SC and other OARs were reported. Especially PA plans seem to show clear dosimetric disadvantages in spinal bone irradiation.^20^

The present study appears to confirm those results on a broader basis due to its significantly larger sample size. Moreover, to the best of our knowledge, this is the first study evaluating a 3D‐CRT technique specially designed to limit SC dose exposure. As shown, 1F2S‐18MV can offer a significant dose limitation to the SC and still deliver sufficient dose coverage of the spinal target volume while maintaining reasonable dose exposure to the other OARs. The overall similar results compared to the original plans might indicate a viable basis for the daily use of this simple algorithmic technique, if local peculiarities (i.e., actual location of all OARs) are taken into account. If desired, further improvements of SC dose limitation using 1F2S‐18MV are easy to achieve (e.g., by stronger weighting of lateral segment beams). Moreover, 1F2S‐18MV does not even necessarily require a planning CT scan, as lateral segment fields could be defined with 2D imaging only (see Figure S2). Our findings also seem to be transferable for use of 6MV photons since the results of 1F2S‐18MV and 1F2S‐6MV were on a comparable level.

It must be emphasized that our study only included cases without gross tumor involvement of the spinal canal. The conclusions drawn should only be applied to this clinical constellation since limiting dose to the spinal canal in case of tumor involvement in this area could counteract the treatment goal.

The dosimetric advantages of the evaluated 3D‐CRT over conventional PA and APPA plans might be estimated conservatively, since also PA and APPA were planned on a 3D (i.e., CT scan) basis. Hence, real 2D planning might be even more inferior.^21^ Nevertheless, besides dosimetric considerations, the pending question remains, whether there is an actual clinical benefit for spinal irradiation by using 3D‐CRT approaches. In a study by Pope et al., a prospective cohort of patients treated with 3D‐CRT plans for bone metastases (50% spine involvement) was compared to a historical cohort treated with 2D plans and no difference in patients‐related outcomes were observed.^22^ Two other retrospective series concerning irradiation of bone and spine metastases however revealed a tendency for better clinical outcomes of 3D‐CRT versus 2D planning in terms of pain response and toxicity.^23^, ^24^ Unfortunately, further evidence from prospective trials is not available, so a definitive conclusion cannot be drawn yet.^25^

When thinking about the appropriate irradiation technique in daily practice, one should also be concerned by the possible need for future re‐treatment of the same spinal site. For a long time, radiation oncologists feared re‐irradiation of the spine due to the increased risk of radiation‐induced myelopathy. However, several studies have demonstrated the effectiveness and feasibility of a second or even third course of spinal RT with low rates of neurological complications.^26^, ^27^, ^28^, ^29^, ^30^, ^31^ The response to irradiation persists for a few months only.^32^ Hence, for patients whose life expectancy exceeds this period of time, considering the possibility of a required re‐irradiation should be of importance. It seems obvious that for this relevant cohort of patients controlled dosimetric conditions in the initial treatment situation are desirable. Thus, in this setting, the utilization of RT techniques should be considered that deliver sufficient dose coverage to the target volume and limit SC doses without exceeding dose load of other OARs. Demonstrating the practicability of such a technique might be the main result of this study. We therefore believe that 1F2S‐18MV is a viable basis for RT treatment planning of spinal metastases, in particular for patients with more advantageous survival prognosis who potentially require re‐treatment in the course.

## 5 CONCLUSION

Our data strengthen the assumption that 3D‐CRT techniques in treatment planning of spinal metastases have a clear dosimetric and potential clinical advantage over conventional 2D techniques. Simple 3D‐CRT variants like 1F2S‐18MV can additionally offer a significant dose limitation to the SC while providing a sufficient dose coverage of the target volume. Such 3D‐CRT approaches could therefore be a rational treatment option for patients with favorable life expectancy and potential need for future re‐irradiation.

## AUTHOR CONTRIBUTIONS

Developed study setup: Christian Cornelius Arnold, André Toussaint, Klaus Bratengeier; conducted radiation treatment planning: Christian Cornelius Arnold; analyzed data: Christian Cornelius Arnold, Klaus Bratengeier; wrote the manuscript: Christian Cornelius Arnold, André Toussaint, Frederick Mantel, Klaus Bratengeier. All authors contributed to the review of the manuscript and all approved the final draft for submission.

## CONFLICT OF INTEREST STATEMENT

The authors declare no conflicts of interest.

## Supporting information

Supporting Information

Supporting Information

## Data Availability

The datasets used and/or analyzed during the current study are available from the corresponding author on reasonable request.
