# Supplementary material for: Dosimetric evaluation of a spinal cord dose‐limiting 3D‐CRT technique for radiotherapy of spinal metastases
Source: J Appl Clin Med Phys. 2023 Sep 7;24(10):e14042. doi: 10.1002/acm2.14042 (PMC10562034; doi:10.1002/acm2.14042)
Supplement: Supplementary file 1 — Supporting Information [file ACM2-24-e14042-s002.docx]

**Additional Figures**

**Additional Fig. 1. Distribution of target volumes in relation to the spine.** Y-axis: numbering of the 41 target volumes. Upper x-axis: vertebrae from cranial (C1) to caudal (L5). The yellow boxes represent the vertebrae implemented in each target volume. Lower x-axis: number of appearances of each vertebra.

**Additional Fig. 2. Optional 2D treatment planning for 1F2S-18MV segment fields.** Imaging for conventional radiation treatment planning exemplarily represented by digitally reconstructed radiographs (DRRs): Beam’s eye view with a gantry angle of 245° (a), with MLC adaption to the rear edge of the vertebral body (b) and additional visualization of the spinal canal in green (c and d).

**Data Availability Statement**

The datasets used and/or analyzed during the current study are available from the corresponding author on reasonable request.
