# Supplementary material for: Dosimetric evaluation of a spinal cord dose‐limiting 3D‐CRT technique for radiotherapy of spinal metastases
Source: J Appl Clin Med Phys. 2023 Sep 7;24(10):e14042. doi: 10.1002/acm2.14042 (PMC10562034; doi:10.1002/acm2.14042)
Supplement: Supplementary file 2 — Supporting Information [file ACM2-24-e14042-s001.docx]

**Additional Table 1. Estimated treatment plan delivery time.** Due to the inconsistent number of angles and segments, the formula* was not applied to the original plans.

| **Technique** | **1F2S-18MV** | **PA** | **APPA** | **1F4S** | **WD** | **1F2S-6MV** |
| --- | --- | --- | --- | --- | --- | --- |
| **Treatment delivery time [seconds]** | 103 | 42 | 76 | 113 | 150 | 114 |

*as described by Bratengeier et al., Radiat Oncol, 2011:

The plan delivery time *T*depends on

1) the number of equidistant gantry angles *n*;

2) the number of segments *N*;

3) the gantry rotation time between beams *τ_GSS_*. The gantry rotation time includes the start-stop time of the gantry *τ_SS_*, and the gantry rotation speed per degree *v_G_*:

𝜏𝐺𝑆𝑆=(𝜏𝑆𝑆+360∘𝑣𝐺);

4) the segment shaping time, which depends strongly on the leaf speed and the changes in the segment shapes between segments. For the sake of simplicity the mean segment shaping time, 𝜏⎯⎯𝑆, was considered. Segment shaping and gantry positioning are simultaneous, so the more time consuming process dominates;

5) the pure irradiation time *MU*/
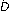
, where *MU*is the total number of monitor units, 
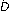
 is the dose rate [MU/min];

6) the data handling time per beam *τ_F_*:

𝑇≈(𝑛−1)⋅𝑀𝑎𝑥{𝜏𝐺𝑆𝑆,𝜏⎯⎯𝑆}+(𝑁−𝑛+1)⋅𝜏⎯⎯𝑆+𝑀𝑈*Ḋ*+(𝑛−1)⋅𝜏𝐹

*T*was estimated using measured parameters: *v_G_*= 60 s/360°, *τ_SS_*= 3 s, 𝜏⎯⎯𝑆 = 7 s, *τ_F_*= 3 s.
